# Sustained Magnetic Responses in Temporal Cortex Reflect Instantaneous Significance of Approaching and Receding Sounds

**DOI:** 10.1371/journal.pone.0134060

**Published:** 2015-07-30

**Authors:** Dominik R. Bach, Nicholas Furl, Gareth Barnes, Raymond J. Dolan

**Affiliations:** 1 Wellcome Trust Centre for Neuroimaging, University College London, London, United Kingdom; 2 Department of Psychiatry, Psychotherapy, and Psychosomatics, University of Zurich, Zurich, Switzerland; 3 MRC Cognition and Brain Sciences Unit, University of Cambridge, Cambridge, United Kingdom; UNLV, UNITED STATES

## Abstract

Rising sound intensity often signals an approaching sound source and can serve as a powerful warning cue, eliciting phasic attention, perception biases and emotional responses. How the evaluation of approaching sounds unfolds over time remains elusive. Here, we capitalised on the temporal resolution of magnetoencephalograpy (MEG) to investigate in humans a dynamic encoding of perceiving approaching and receding sounds. We compared magnetic responses to intensity envelopes of complex sounds to those of white noise sounds, in which intensity change is not perceived as approaching. Sustained magnetic fields over temporal sensors tracked intensity change in complex sounds in an approximately linear fashion, an effect not seen for intensity change in white noise sounds, or for overall intensity. Hence, these fields are likely to track approach/recession, but not the apparent (instantaneous) distance of the sound source, or its intensity as such. As a likely source of this activity, the bilateral inferior temporal gyrus and right temporo-parietal junction emerged. Our results indicate that discrete temporal cortical areas parametrically encode behavioural significance in moving sound sources where the signal unfolded in a manner reminiscent of evidence accumulation. This may help an understanding of how acoustic percepts are evaluated as behaviourally relevant, where our results highlight a crucial role of cortical areas.

## Introduction

Rising sound intensity is a potent warning cue for humans [1–3] and other primates [4], probably because it is the key motion cue signalling approach ("looming") of sound sources. Indeed, orienting responses to rising intensity and to approaching sound sources are comparable [5]. Compared to falling intensity, rising intensity elicits a stronger orienting response in humans [1, 5, 6], increased phasic alertness within and across modalities in humans and monkeys [1, 5, 7–9], as well as a perceptual bias towards intensity change [2, 5] and perceived sound source distance [3, 10, 11] in humans. The behavioural significance of approaching sound sources (as compared to receding ones) is underlined by the fact that rising intensity and approaching sounds are explicitly rated in humans as more negative, more activating, more intense, more significant, more threatening, and elicit stronger feelings of unpleasantness and activation compared to falling intensity or receding sounds [5, 6]. How this evaluation as behaviourally significant is instantiated neurally, however, remains an open question.

Human functional resonance magnetic imaging [fMRI] studies have demonstrated hemodynamic responses to rising versus falling intensity in auditory areas encompassing temporal plane, superior temporal sulcus and middle temporal gyrus, and in the amygdala [1, 12]. These responses are viewed as reflecting the perception of an approaching versus a receding sound source, and its evaluation as behaviourally significant. The evolution of this percept, and of its appraisal, is below the temporal resolution of fMRI. Neurophysiological studies in rhesus monkeys, on the other hand, have revealed that neural activity in lateral belt auditory cortex is stronger for looming than for receding sounds [13], and that this activity synchronises with visual cortex activity during perception of audio-visual looming sounds [14]. These findings make the auditory cortex one prime candidate for generation of a percept that encodes the behavioural significance of these sounds.

In the present study, we were interested in the temporal evolution of this percept. To this end, we investigated in humans the time course of approaching sound perception with magnetoencephalography [MEG]. As in previous studies, we used rising and falling intensity as cue for approach/recession. The behavioural significance of rising and falling intensity in humans and monkeys as well as neural responses in auditory belt area of monkeys relies on complex (i. e. non white-noise) sounds [2, 3, 7, 13]. As a likely reason, it has been suggested that intensity change in white noise sounds is not perceived as approach or recession [3]. In fact, for physically approaching sound sources, the intensity change over time depends on the sound frequency; a uniformly rising intensity in white noise sounds renders them impossible to reflect an approaching sound source [15]. A behavioural asymmetry was found between white-noise and different complex sounds, including a defined but limited frequency spectrum [13], triangular waveforms [3, 7], or synthetic vowel sounds [2, 3] and hence does not rely on specific properties of the complex sounds. By comparing intensity envelopes of complex and white noise sounds in the present study, we were able to discard responses to rising intensity per se (occuring to both types of sound) and focus on specific responses to approaching and receding sound sources (occurring only to complex sound). Finally, to disambiguate responses to apparent instantaneous distance (corresponding to instantaneous intensity in complex sounds) from responses to behaviourally significant apparent distance change, we used two levels of overall intensity, which differ, at each point in time, in terms of instantaneous intensity, but not intensity change.

We hypothesised that auditory association areas would encode behavioural significance for an approaching or receding sound, over and above encoding intensity change, or apparent distance. We term the sound on which the intensity envelope is imposed a carrier sound. Thus, our focus was on an interaction of *intensity change x carrier sound*, which was not duplicated in an interaction of *overall intensity x carrier sound*.

## Materials and Methods

### Design and participants

The study followed a 2 × 2 × 2 factorial design (Table 1) with the factors *intensity change* (rising, falling), *carrier sound* (complex tone, white noise), and *overall intensity* (59 dB [50–68 dB], 77 dB [68–86 dB]). We recruited 23 healthy individuals (12 male, 11 female, mean age ± standard deviation, 23.0 ± 3.7 years) from the general population via advertisements at University College London. Three participants had more than 20% artefact trials and were excluded from analysis. All participants were adults who gave written informed consent, and were fully informed about the aims of the study. The study protocol, including the form of taking written informed consent from participants, followed the principles expressed in the Declaration of Helsinki, and was approved by the National Hospital for Neurology and Neurosurgery and Institute of Neurology Joint Research Ethics Committee.

**Table 1:** Study design. Italics indicate the interaction of primary interest.

2x2x2 Factorial design		Intensity change			
		Rising		Falling	
**Carrier frequency**	**Complex tone**	**High**	**Low**	***High***	***Low***
		50–68 dB	68–86 dB	*68–50 dB*	*86–68 dB*
	**White Noise**	***High***	***Low***	**High**	**Low**
		*50–68 dB*	*68–86 dB*	68–50 dB	86–68 dB

### Independent variables and sound stimuli

**Fig 1:**
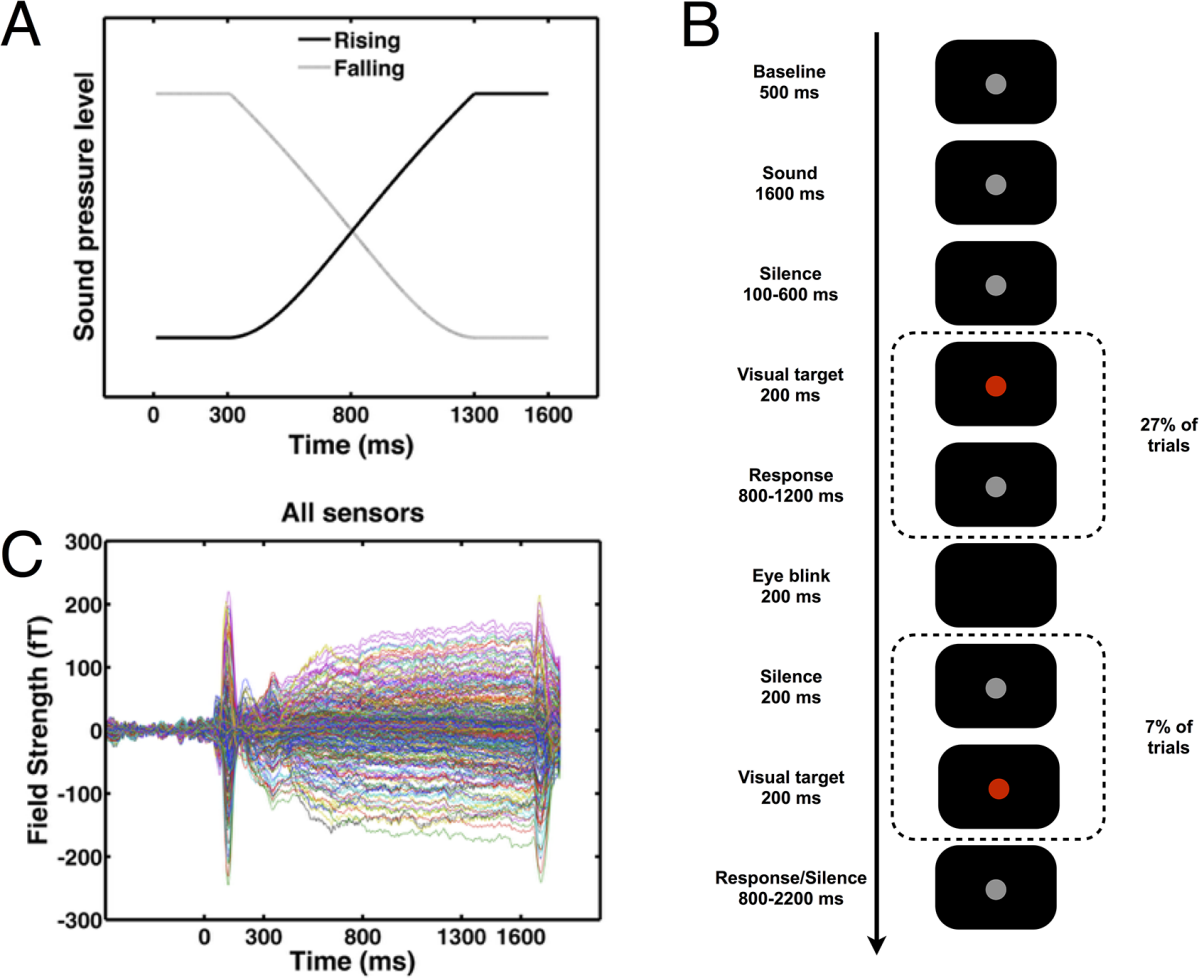
Experimental procedures. A: Illustration of the sound stimuli used in this experiment. After an initial constant sound pressure level [SPL] segment of 300 ms, SPL rises (or falls) over 1000 ms; the sound terminates with another segment of constant SPL. B: Intra-trial procedure. Our analysis focuses on responses to the sound. Participants were instructed to blink only in designated periods between sounds. A visual task was introduced between sound presentations to increase alertness and render the eye blink task plausible to the participant. C: Global sound responses to all sound stimuli, for all sensors. The intensity change segment of the sound was between 300 and 1300 ms.

Amplitude modulated carrier tones were presented dichotically, with sound amplitude adjusted individually for each ear, via a pneumatic system and ear moulds (Etymotic Research, Elk Grove Village IL, USA). Intensity was quantified as sound pressure level [SPL], expressed in dB. After an initial 300 ms with constant amplitude, amplitude rose quadratically over 1000 ms to the terminal intensity, which was then maintained for another 300 ms in order to disambiguate responses to on/offset from responses to amplitude modulation. A quadratic amplitude envelope was chosen in keeping with a previous study [13]. Over the amplitude region chosen, the SPL envelope was close to linear (Fig 1A). Rising and falling sounds were identical, with falling sounds being reversed in time. Carrier tones were 50% duty cycle square wave sounds with 440 Hz fundamental frequency ("complex sounds"), and uniform broadband white noise sounds ("white noise sounds") with spectral constraints imposed by stimulus duration and sampling frequency (i. e. 0.6 Hz—22 kHz). Mean intensity was 59 dB [50–68 dB], and 77 dB [68–86 dB] respectively, in line with a previous study [13]. SPL were established independently for both earphones using a 2 cm^3^ ear simulator (Type 4157 with Two Channel Microphone Power Supply Type 2807; Brüel & Kjær; Nærum; Denmark) and an online power analyzer (Portable Dual Channel FFT Analyzer CF350Z; Ono Sokki; Yokohama; Japan); the simulator was calibrated using a 250 Hz 124 dB sine wave (generated by Pistonphone Type 4220; Brüel & Kjær; Nærum; Denmark). Since subjective loudness perception might not be completely explained by objective SPL, we used a matching procedure after the main experiment to compare complex sounds with white noise sounds, and to compare loudness perception between left and right ear. Each of the two carrier sounds was presented dichotically with static SPL of 50 dB, 68 dB, and 86 dB for three times, summing up to 18 trials in randomised order. This was the fixed sound, and participants were tasked to adjust the respective other carrier sound to have the same subjective loudness, using a vertical lever without descriptions that represented SPL from 35 to 95 dB. Initial SPL for this adjustable sound was determined randomly on each trial. Participants could hear the fixed and the adjustable sound as often as they wanted, and had to press a confirm key to proceed to the next trial. Across participants, there was no difference between adjusted SPL for complex and white noise sounds. Compared to fixed white noise sounds with 68 dB average SPL, complex sounds were adjusted to 68.5 ± 0.4 dB. Compared to fixed complex sounds, white noise sounds were adjusted to 67.5 ± 0.3 dB (paired t-test, t(19) = 1.6, p = .12). Following this, each of the two carrier sounds was presented with each of three SPL (50 dB, 68 dB, 86 dB) on each ear separately for two times, summing up to 24 trials in randomised order. Participants were tasked to adjust the same carrier sound on the other ear to have the same subjective loudness, using the procedure described above. Across the group, all sounds were perceived as louder on the left than on the right ear (adjusted SPL left: 69.5 ± 0.7 dB; adjusted SPL right: 66.1 ± 0.8 dB; paired t-test, t(19) = 2.7, p < .01).

### Procedure

Upon arrival at the laboratory, the procedure was explained to participants. Participants were then positioned in the MEG and engaged in a practice without sound stimuli to habituate to eye blink and key press tasks. The actual experiment then commenced, and was divided into 5 blocks of 120 trials (approximately 8 minutes), i. e. 600 trials overall. After finishing the MEG recordings, the sound matching tasks were performed as described above.

### Intra-trial procedure

During trials, a dark grey fixation disk (20% grey) was present in the lower half of the screen. Participants were tasked to fixate and blink only during a designated eye blink period during which the fixation disk disappeared. Each trial started with a baseline period of 500 ms (Fig 1B). A sound stimulus was then presented for 1600 ms, followed by a silent interval of at least 100 ms, possible visual target, and an eye blink period. The visual task was introduced similar to previous studies [1, 5] as an active element to keep participants attentive in this passive listening task. After the eye blink period or the last visual target, there was a silent interval of at least 800 ms before the next baseline period started.

For the between-trial time, there were 3 different types:

1. No visual target: For 440 trials (73%, or 11 per sound type per session), the sound stimulus was followed with equal frequency by a silent interval of 100, 200, or 300 ms. During a subsequent eye blink period of 200 ms, the fixation disk disappeared. Participants were instructed to only blink during these periods, although they did not have to make use of every eye blink period. After the eye blink period, another silent period followed that was, at equal frequency, 900, 1100, or 1300 ms in length, minus the deviation of the pre-blink period from 200 ms (that is, +100, 0, or -100 ms). Thus, the minimum post-blink period was 800 ms before the next baseline period started.
2. One visual target: For 120 trials (20%, or 3 trials per sound type per session), the sound stimulus was followed with equal frequency by a silent interval of 200, 400, or 600 ms, to estimate the time course of visual phasic alertness. The fixation disk then turned red for 200 ms, and participants had to press a response key as quickly as possible. If they did not respond within 800 ms, a warning message appeared for 1000 ms. The response period was followed by a silent interval and eye blink period as described above.
3. Two visual targets: For 40 trials (7%, or 1 per sound stimulus per session), the sound stimulus was followed by a silent interval of 200 ms. Then a visual target followed as described above. After the response period of 800 ms, a silent interval of 400 ms was followed by an eye blink period of 200 ms and another 200 ms silent interval. Then a second target appeared for 200 ms, again followed by a response period of 800 ms, and a silent interval of, at equal frequency, 900, 1100, or 1300 ms. This condition was introduced to make the eye blink period plausible–if participants blinked too late, they would risk missing a second visual target.

Responses to all targets were analysed in a 2 (*intensity change*) x 2 (*carrier sound*) x 2 (*overall intensity)* x 4 (*target latency*: 200 ms, 400 ms, 600 ms, second target) ANOVA.

### MEG recordings

MEG recordings were made in a magnetically shielded room (MSR) by using a 275-channel CTF system with SQUID-based axial third order gradiometers (VSM MedTech Ltd) with a hardware anti-alias low pass filter of 300 Hz cut off frequency and sampling rate of 1200 Hz. No high pass filtering was applied. Participants made responses with an MEG-compatible response pad, held in the right hand. Visual stimuli were projected from outside the MSR onto a screen in front of the participant. Fiducial measurements (nasion and 1 cm anterior of tragus one each side) were made using the manufacturer's procedure. Eye blinks were monitored using an EyeLink 1000 eye tracker (SR Research Ltd), and the analogue output was recorded together with the MEG channels.

### MEG analysis

MEG data was analysed using standard procedures in statistical parametric mapping (SPM 8; Wellcome Trust Centre for Neuroimaging, London, UK; *http*:*//www*.*fil*.*ion*.*ucl*.*ac*.*uk/spm*). The time series was first subjected to an initial artefact correction to detect spikes and sudden jumps due to squid resetting, defined by a signal change between two data points exceeding 3000 fT. For these artefacts we used a median filter over 20 data points to correct the derivative of the signal, and then reconstructed the time series from the derivative. Trials were epoched from 500 ms before sound stimulus onset until 1800 ms after sound onset, corresponding to 200 ms after sound offset. Eye blinks were identified from the eye tracker and defined as loss of pupil size tracking. All epochs containing spikes, squid resettings, eye blinks or MEG channel values exceeding a threshold of 3000 fT, were rejected as artefacts. Between 5.0% and 12.3% of trials were excluded due to artifacts (mean: 6.7%). Data were then merged across blocks, averaged within conditions, low-pass filtered with a first-order Butterworth filter and cut off frequency of 80 Hz, and down sampled to 200 Hz. Analysis included two steps: (1) To account for the multiple comparisons across sensors and time, we chose an imaging approach for analysis of sustained fields on the sensor level. Data were interpolated across the scalp and written out into 3-D image (scalp x time) volumes with 32x32 voxels in scalp space, each voxel corresponding to 4.25x5.38 mm of surface space. This procedure accounts for the actual position of the head within the dewar by using fiducial measurements. Thus, the same voxel in each dataset corresponds to the same position of the head surface rather than to the same sensor. Images were smoothed with a Gaussian filter with a full width at half maximum (FWHM) of 40 mm in space, and 80 ms in time, to account for anatomical variability. Contrasts of interest were generated and tested for consistency on the group level using one-sample t-tests. P-values were whole-volume corrected for family-wise error using Gaussian random field theory [16], and are reported at a threshold of p < .05 corrected on the cluster level. (2) To localise the magnetic source activity generating the impact of intensity change in complex as opposed to white noise sounds, we inverted the time series of the contrast (Rising > Falling) > (Complex > White Noise) for each individual dataset, and in post-hoc tests the simple main effects (Complex > White Noise) in Rising, and (Complex > White Noise) in Falling. To this end, we used the imaging solution implemented in SPM. We employed a single shell forward head model with canonical mesh (2 mm resolution), co-registered to the subject data. The model was inverted with multiple sparse priors and group inversion [17]. To improve estimability, sources were broadly restricted to auditory areas. This was achieved by placing spherical regions of interest with 32 mm radius along the lateral fissure and superior temporal sulcus. The outer spheres were located at MNI coordinates ±50/8/-30 and ±50/-48/3 for the STS and ±50/20/-13 and ±50/-44/23 for the lateral fissure; in-between we placed 3 more equidistant spheres along each structure. The resulting region of interest encompassed the entire temporal cortex and neighbouring frontal and parietal cortices, and thus all structures previously described in fMRI studies to be involved in perception of looming sounds [1, 12]. Estimated source power in the frequency band 0–1 Hz, reflecting slow changes of the sustained field, were averaged over the time window of the intensity change (300–1300 ms) and written out into 3D images. These were smoothed with an 8 mm FWHM Gaussian filter, and tested for consistency across the group using one-sample t-tests. To account for multiple comparisons, results were cluster-level corrected for family wise error, and are reported at a voxel-selection threshold of p < .001 and a corrected threshold of p < .05.

## Results

### Event-related fields

Sound onset evoked magnetic fields with separable components over the first ~300 ms (see Fig 1C). Sustained fields then showed an evolution over time during the ramp of intensity change which lasted until 1300 ms after sound onset. After 1600 ms, the sound ended, and an offset response was observed.

ERFs were statistically analysed in a random field theory based approach that accounts for multiple comparisons across space and time [16]. Across the group, we observed main effects of *intensity change* (Rising vs. Falling), *overall intensity* (High vs. Low) and *carrier frequency* (Complex vs. White Noise), both during the onset response, and during the sustained response; these were not the main focus of this report and are not further analysed. Importantly, there was a significant interaction of *intensity change* (Rising vs. Falling) with *carrier frequency* (Complex vs. white noise) in sustained responses over bilateral temporal sensors, extending between ~900 ms and ~1400 ms after sound onset (Table 2). As hypothesised, this indicates responses to apparently approaching/receding sound sources.

**Table 2:** Inference statistics for the interaction intensity change (rising vs. falling) with carrier frequency (complex vs. white noise) of sustained fields (Figs 2 and 3), and for second-level t-tests on source localisation results (Fig 4). All results are significant at a threshold of p < .05, corrected for family wise error at the cluster level across the entire scalp and trial duration, across the scalp, or across the brain volume, respectively. No other results survived whole-brain correction.

Peak t-value	Location	
*Sustained Fields*	*Sensor closest to local maxima across the group*	*Time [ms after sound onset] of local maxima*
7.6	MLT26/MLT36/MLT16	1215/1015/1400
4.9	MRP23/MRO14	1475/1390
*Inversion results*	*Spatial location of local maxima*	*Coordinates [x/y/z mm in MNI space] of local maxima*
9.84	Right middle temporal gyrus/temporo-parietal junction	48/-68/2, 40/-66/0, 44/-68/-12
5.59	Right inferior temporal gyurs	-42/-44/-20, -46/-18/-34, -44/-24/-28
4.08	Left inferior temporal gyrus	54/-22/-30, 56/-32/-26, 48/-16/-36

**Fig 2:**
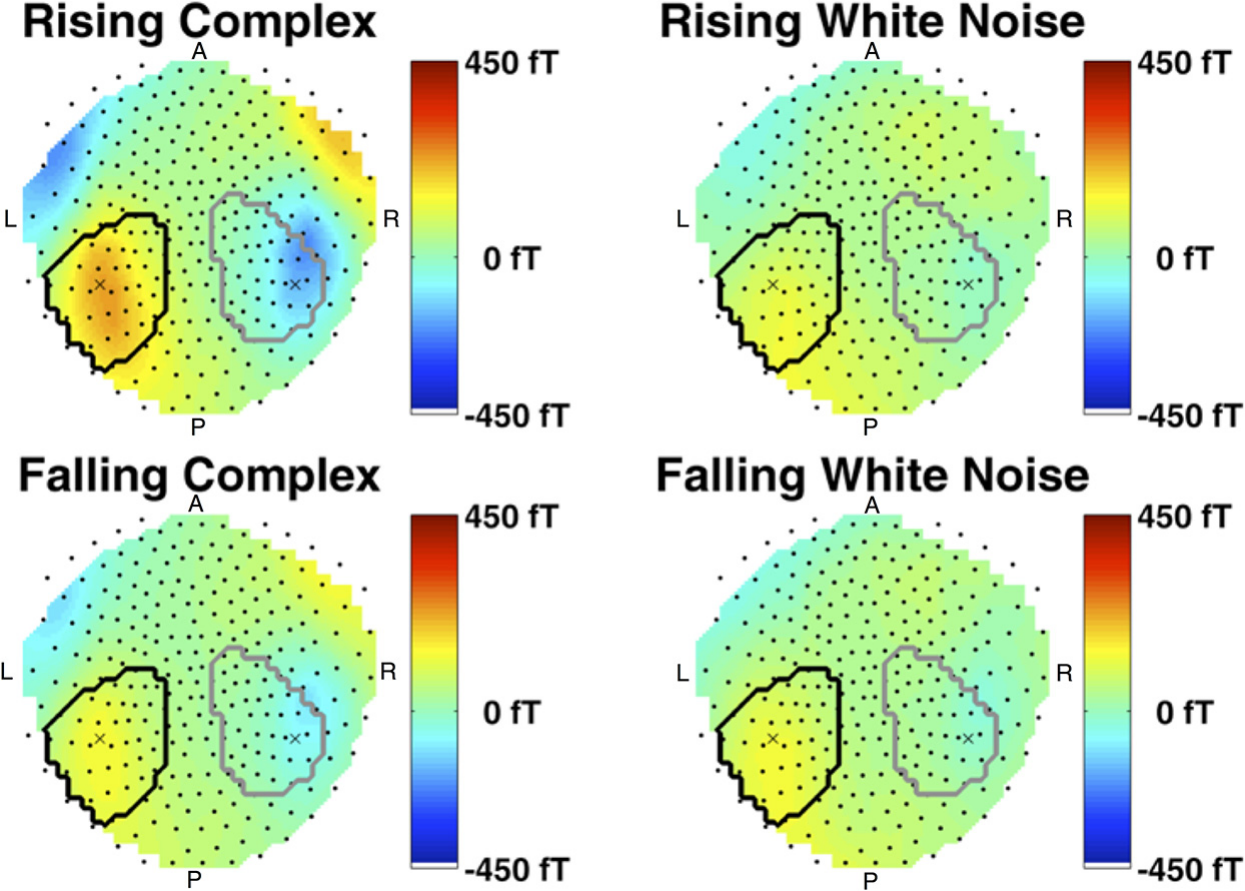
Sustained fields across the scalp. Fields are shown for the most significant time point of the *intensity change x carrier frequency* interaction (1215 ms after sound onset, 915 ms into the intensity change), averaged across the group, for the four different conditions in this interaction. Dots: average sensor positions. X: Most significant point in sensor space and its contralateral counterpart (see Fig 3). Black outline: location of significant left hemispheric cluster at 1215 ms. Grey outline: location of significant right hemispheric cluster at its most significant time point, 1390 ms after sound onset.

**Fig 3:**
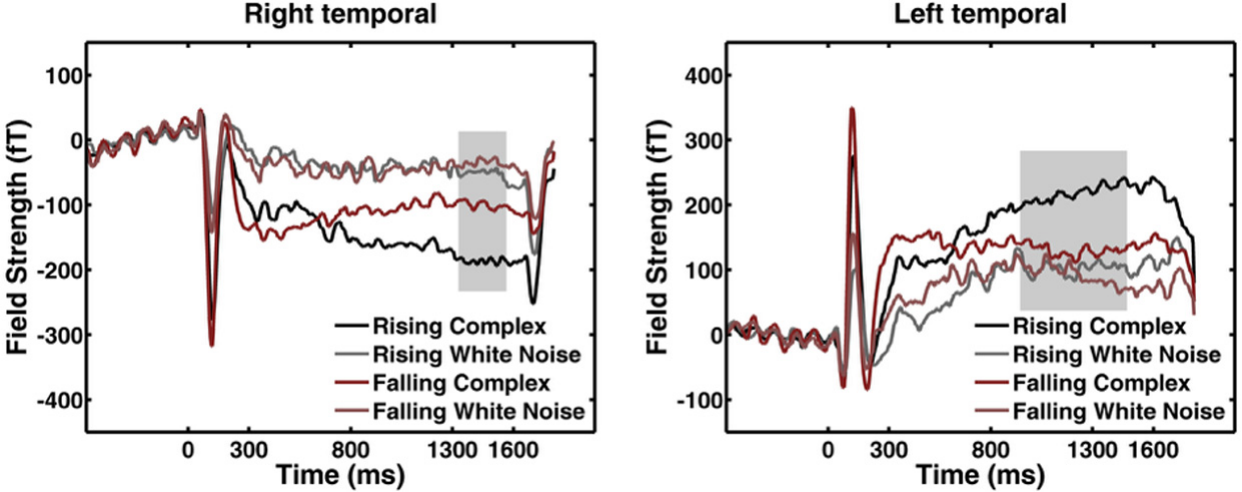
Field time courses. All time courses are shown for the most significant point in sensor space of the *intensity change x carrier frequency* interaction, and its contralateral counterpart, averaged across the group, for the four different conditions in this interaction. Location of this point in space is plotted in Fig 2. Averaged across the group, the closest sensors at these scalp points were MLT26/MRT16. Grey background: significant time points at these points in space.

**Fig 4:**
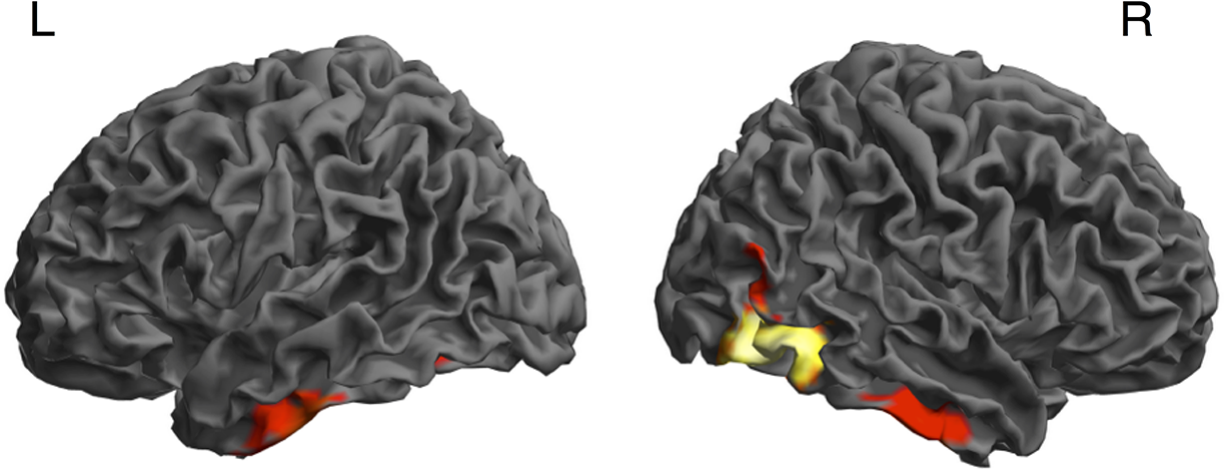
Source-level analysis. Putative sources of the observed *intensity change x carrier frequency* interaction: significant clusters in a group-level t-test on source inversion results of the *intensity change x carrier frequency* interaction (see Table 2).

Fig 2 illustrates the group (grand) average of the ERF interaction intensity change x carrier frequency at its most significant time point during the ramp, i. e. 1215 ms after sound onset and 915 ms into the ramp. Absolute field strength in temporal areas is higher for complex than for white noise sounds, and there is not much difference between the two white noise sounds. For complex sounds, absolute field strength is higher for the rising than for the falling sound at this particular point in time. Fig 3 shows the field time course for the most significant point in scalp space, and its contralateral counterpart. Averaged across the group, the closest sensors to these points were MLT26/MRT16. The plots suggest that absolute field strength of rising and falling complex sounds starts off approximately at the same level (despite different intensity at this point in time) and, across the entire ramp, tracks intensity change of both rising and falling sounds. This does not occur for white noise sounds. Post-hoc tests showed that field strength was different between complex and white noise sounds both when intensity change was rising, and when it was falling.

Formally, the interaction we observed might be due to different behavioural significance or to a different gradient in apparent distance, but also to different instantaneous apparent distance. In other words, different responses to complex rising and falling sounds at a particular point in time might be due to the fact that these appear to be at different distances at this point in time. We used sounds with different overall intensity to exclude this possibility; these sounds differ in apparent distance across the entire trial duration. An *overall intensity* x *carrier sound* interaction would indicate responses that are specific to instantaneous apparent distance of complex sounds. We did not observe any responses in this interaction at a significance level of p < .05. This suggests that the interaction *intensity change x carrier sound* does not depend on differences in instantaneous apparent distance but rather on difference in distance gradient, or behavioural significance. This is also illustrated in Fig 3 which shows that sustained field responses to rising and falling sounds have similar initial strength–despite different apparent distance–which then diverges as the sound progresses.

### Source reconstruction

Finally, we were interested in the source of the sustained fields that track intensity changes for complex but not white noise sounds. The interaction contrast *intensity change x carrier frequency* was inverted, for each participant, for the frequencies between 0 and 1 Hz, and the time window of the ramping sound (300–1300 ms after sound onset), using the MSP algorithm engendered in SPM. Source power was extracted from the 0–1 Hz band using Morlet wavelet projection, and written into individual 3D images. These were then tested for consistency on the group level. This analysis revealed a significant cluster of source activity in right temporo-occipito-parietal junction and two smaller clusters in bilateral inferior temporal gyrus (Table 2, Fig 4).

In post-hoc tests, we then examined the localisation of sources pertaining to rising and falling complex sounds. Complex sounds, compared to white noise sounds, engaged sources in a similar network for rising and falling intensity. This network comprised clusters in the bilateral inferior/middle temporal gyrus, right superior temporal, and left fusiform gyrus. Additionally, it extended into bilateral middle occipital and right inferior occipital gyrus for rising sounds, and into right fusiform and inferior frontal gyrus, right insula, and left superior temporal gyrus, for falling sounds. In other words, the bilateral inferior temporal cluster showing up in the interaction was recruited by both rising and falling sounds, but to a different extent. In contrast, the activated sources extended further posterior into the left temporo-occipital-parietal junction only for rising and not for falling sounds, thus explaining the interaction cluster in this area.

### Responses to visual targets

Reaction times, analysed in a 2 x 2 x 2 x 4 ANOVA, revealed a significant 4-way interaction (F(3, 57) = 3.7; p < .05). Post hoc 2 (*intensity change*) x 2 (*carrier frequency*) ANOVAs for each combination of *overall intensity* and response type showed that 400 ms after sound offset, RTs to visual targets following low *overall intensity* rising sounds were faster than after low *overall intensity* falling sounds, an effect seen only when carrier frequency was complex but not when it was white noise (p < .05). No other significant effects emerged for other response types or for high *overall intensity* sounds.

## Discussion

Rising intensity in complex sounds is perceived as an approaching sound source with behavioural significance. Here, we investigated sustained magnetic fields during perception of such sounds. We show that absolute strength of sustained fields over bilateral temporal sensors linearly track intensity change in complex but not in white noise sounds. At the same time, overall intensity is represented in a similar manner for complex and for white noise sounds. This suggests that sustained fields follow the behavioural significance of an approaching or receding sound source: fields relating to both kind of sound sources have the same initial strength, which then increases for approaching and decreases for receding sounds. These sustained fields do not appear to track apparent distance of the complex sounds, and this is different from what has been suggested for spiking frequency of sensory afferents in fish species which track apparent distance [18].

These findings are in keeping with the spatial hypothesis that auditory cortex generates a percept encoding the behavioural significance of looming sounds, based on previous monkey [13, 14] and human studies [1, 12]. Using source localisation techniques, however, the bilateral inferior temporal gyrus emerged as the most likely source for this sustained field. This area is not considered part of the auditory cortex. On the other hand, both rising and falling complex sounds in our study engaged sources across the entire temporal lobe, including auditory areas. While there is some spatial uncertainty in MEG source reconstruction, possibly explaining localisation differences between our and previous studies, it is also the case that sustained fields in our study, and fMRI responses as well as direct neural recordings in previous work, relate to different stages of processing. We observed that sustained fields track instantaneous behavioural significance–both rising and falling sounds start with almost the same absolute field strength which then linearly increased for rising and linearly decreases for falling sounds. BOLD responses in previous studies [1, 12] sum neural activity over time and are more likely to contain additional summary evaluations of these sounds. Similarly, direct neural recordings from rhesus monkey auditory cortex shows no modulation of looming-induced signals over time [7, 13]. Thus, a possible conclusion from our study that a behavioural significance evaluation emerges outside the auditory cortex does not contradict previous work. The inferior temporal gyrus is in close neighbourhood to other areas implicated in perception/evaluation of approaching vs. receding object motion [1, 12, 19], namely the superior and middle temporal sulcus, and middle frontal gyrus. It has also been implicated in visual perception within what is referred to as the ventral visual pathway [20]. Given that approaching objects are commonly characterised by a combination of auditory and visual cues [7, 21, 22], an involvement of the visual system in providing a rich representation of the likely behavioural significance of approaching sounds provides a plausible interpretation of our data, and links our findings to previous cross-modal investigations [21–25]. This interpretation can also account for an additional and stronger magnetic source detected in the right temporo-parietal junction (TPJ). BOLD signal in the right temporo-parietal junction has previously been observed to reflect in looming sound perception [12]. Among other functions, the right-hemispheric temporo-parietal junction in particular has been implicated in bottom-up (stimulus-driven) attention, together with the inferior frontal cortex [26]. This would be consistent with the behavioural propensity of looming sounds to increase phasic attention within and across modalities. Relating to this interpretation of temporo-parietal source activity, we observed an impact of sounds on reaction times to a subsequent visual target, indicating that phasic alertness was increased 400 ms after the sound offset for rising vs. falling complex, but not white noise sounds. Phasic alertness after rising vs. falling complex sounds has been reported previously for auditory targets [1, 5] 100 ms after sound offset, while these studies are inconsistent with respect to visual targets–one reporting a decrease [1] and the other an increase in phasic alertness to visual targets [5]. The present study replicates the latter albeit with a different time course. A factor possibly important for cross-modal phasic alertness is the overall loudness of the sounds. We found increased visual alertness only for the low-intensity sounds. This is similar to the fact that lower unisensory stimulation often gives rise to stronger multisensory integration (i.e. an inverse effectiveness) [27]. It would be interesting in future studies to investigate how cross-modal alertness relates to magnetic responses; the low number of visual response trials in our study precluded such analysis.

Our findings bear on the more general question of how an evaluation of behavioural relevance arises from sensory percepts. A previous literature has primarily linked subcortical structures, such as the amygdala, to evaluation of behavioural relevance [28, 29]. In these models, subcortical inputs to the amygdala are of crucial importance [30]. Our MEG study was not designed to demonstrate magnetic activity from deep sources. However, we demonstrate that sensory cortical areas encode such evaluations, too. Crucially, this signal unfolds over time in the second range, reminiscent of sensory evidence accumulation in parietal cortex [31] rather than representing a summary evaluation. This may imply that the evaluation actually takes place in cortical areas, where evidence of behavioural significance might be gathered. This finding adds to a growing literature which puts the computational capacities of sensory cortices centre stage for coordinating defensive responses. For example, contrary to a view that cortical areas are not required for learning a prediction of proximal threat from auditory precursors [30], more recent investigations have called this into question by demonstrating that primary and higher auditory cortices are required if these auditory precursors are naturally occurring sounds [32, 33]. Auditory looming is a model example for behaviourally significant sounds encoding possible threat rather than proximal threat, and our results may suggest a similar structure of the evaluation process.

Although there was no clear lateralization of responses in our initial sensor level analysis (Fig 3), we note that there is some asymmetry in the source estimates with a predominantly right hemisphere source at the TPJ. Such lateralisation has been previously observed in fMRI responses in the TPJ [12].

Numerous neuroimaging studies have addressed the neural representation of intensity (see for an overview, [34]), and have revealed that hemodynamic responses in auditory cortex responses relate to intensity. We also observed overall intensity responses over temporal sensors; however we did not analyse differences between intensity levels further as this was not the focus of the present investigation.

Our paradigm crucially rests on an assumption that intensity change in white noise sounds is not perceived as approaching or receding, and that instantaneous intensity in white noise sounds is not informative about sound source distance. These assumptions are based on previous literature [3, 15] but were not established de novo in the present study. In fact, due to their different frequency spectrum, complex and white noise sounds in our study are possibly distinguished by a different loudness time course even though SPL, and initial/terminal loudness, were matched [15]. Also, perceived urgency (i.e. behavioural significance) of sounds depends on their spectral characteristics [35], and this could possibly be independent from their propensity to generate the percept of an approaching/receding sound source. Investigating these prossibilities, as well as establishing on-line measure of apparent sound distance, would be desirable for future studies.

To summarise, our data suggest an encoding of changing sound source distance, and thus behavioural relevance, is implemented within temporal cortical areas, with the inferior temporal gyrus as a likely candidate source for the observed bilateral signal, and an additional source in temporo-parietal junction. The signal unfolds over time in a manner reminiscent of evidence accumulation processes, and this may indicate a role of cortical areas in the relevance evaluation of sounds.
